# Development of MyREADY Transition BBD Mobile App, a Health Intervention Technology Platform, to Improve Care Transition for Youth With Brain-Based Disabilities: User-Centered Design Approach

**DOI:** 10.2196/51606

**Published:** 2024-10-01

**Authors:** Ariane Marelli, Ronen Rozenblum, Clara Bolster-Foucault, Alicia Via-Dufresne Ley, Noemie Maynard, Khush Amaria, Barb Galuppi, Sonya Strohm, Linda Nguyen, Claire Dawe-McCord, Connie Putterman, Adrienne H Kovacs, Jan Willem Gorter

**Affiliations:** 1 McGill Adult Unit for Congenital Heart Disease Excellence McGill University Health Centre Montreal, QC Canada; 2 Cardiovascular Health Across the Lifespan Program Research Institute of the McGill University Health Centre Montreal, QC Canada; 3 Brigham and Women's Hospital and Harvard Medical School Boston, MA United States; 4 Department of Epidemiology, Biostatistics, and Occupational Health McGill University Montreal, QC Canada; 5 Department of Medicine McGill University Montreal, QC Canada; 6 Department of Psychology Division of Adolescent Medicine The Hospital for Sick Children Toronto, ON Canada; 7 Department of Pediatrics McMaster University Hamilton, ON Canada; 8 CanChild Centre for Childhood Disability Research School of Rehabilitation Science McMaster University Hamilton, ON Canada; 9 Cumming School of Medicine University of Calgary Calgary, AB Canada; 10 Centre for Addition and Mental Health McMaster University Hamilton, ON Canada; 11 Equilibria Psychological Health Toronto, ON Canada

**Keywords:** patient-centered care, patient engagement, mobile app, health IT, health care transition, mobile phone

## Abstract

**Background:**

Transition from pediatric to adult health care varies and is resource intensive. Patient-centered health information technology (HIT) interventions are increasingly being developed in partnership with patients.

**Objective:**

This study aims to develop an internet-based mobile app intervention for patients with brain-based disabilities to improve transition in care readiness.

**Methods:**

The app was designed for patients aged 15 to 17 years with brain-based disabilities having the ability to use a mobile app. A multidisciplinary team, an industry partner, and a patient and family advisory council was assembled. We hypothesized that existing tools could be migrated into the app to address education, empowerment, and navigation. We used cognitive learning theory to support chapters targeting transition in care skill sets. We used the agile iterative methodology to engage stakeholders.

**Results:**

We developed a novel MyREADY Transition HIT platform. An electronic mentor supported cognitive learning with messaging, quizzes, rewards, and videos. We used gaming to guide navigation through a fictitious health care city. Adapting existing tools was achieved by the patient and family advisory council requesting personalization. Our iterative design required time-consuming back-end technology management. Developing the platform took 24 months instead of our grant-approved 12 months, impacting the onset of the planned trial within the allotted budget.

**Conclusions:**

A novel patient-centered HIT platform to improve health care transition was successfully developed in partnership with patients and industry. Careful resource management was needed to achieve timely delivery of the end product, flagging the cautious planning required to deliver HIT tools in time for the much-needed trials informing their clinical application.

**Trial Registration:**

ClinicalTrials.gov NCT03852550; https://clinicaltrials.gov/study/NCT03852550

## Introduction

### Background

A growing number of adolescents with pediatric-onset health conditions have survived to adulthood owing to advances in medical care [1,2]. The Society of Adolescent Medicine defines *health care transition* as “a purposeful, planned process that addresses the medical, psychosocial and educational/vocational needs of adolescents and young adults with chronic physical and medical conditions as they move from child-centered to adult-oriented health care systems” [3-6]. Despite published guidelines, the resource-intensive process of transition in care at the point of care varies widely between institutions and health care systems [2,3,7,8].

Transition in care is largely policy-driven rather than patient-centered [9,10]. Variably between jurisdictions, transition in care is usually mandated to occur when a patient is aged between 18 and 21 years, irrespective of transition readiness [11-13]. However, among youth with chronic conditions, age correlates poorly with transition readiness [14]. Similar to other chronic conditions, brain-based disabilities (BBDs) such as autism, cerebral palsy, epilepsy, fetal alcohol spectrum disorder, and spina bifida require transition in care interventions to improve their outcomes [8]. Youth with BBD report barriers to transition in care, including inadequate support, information, and preparation, resulting in difficulty in navigating adult care systems [3,8,15,16].

Although there are various definitions of patient-centered care, core concepts uniformly highlight the central role that patients and families should play in health care delivery. The Institute of Medicine defines patient-centered care as “care that is respectful and responsive to individual patient preferences, needs and values, and ensuring that patient values guide all clinical decisions” [17]. Systematically, elements include respect for patient preferences, psychosocial support, information, education, and access. Patient-facing mobile apps offer a promising approach to supporting health care transition by educating, engaging, and empowering youth and their families to manage their own health in collaboration with their health care providers [18].

### Objective

The Readiness in Youth for Transition Out of Pediatric Care (READYorNot) BBD trial will test a health information technology (HIT) intervention in a randomized controlled trial (RCT) that aims to improve transition readiness in youth with BBD [19]. In this study, we describe the development of the mobile app that serves as the intervention in the READYorNot BBD trial [19]. The app was created to deliver educational content through an internet-based gamified interface, designed to engage and empower youth with BBD to navigate health care transition. Adolescents with chronic diseases have been shown to benefit from strategies that promote independence with health care management [12]. In line with these findings*,* our goal was to develop an internet-based patient-facing and patient-centered mobile app for youth with BBD to improve transition readiness. We report the design approach of partnering with patients and industry to develop a novel patient-centered HIT platform to improve health care transition. We targeted 3 cognitive pillars of transition in care: education, empowerment, and health care system navigation. In this paper, we illustrate (1) methodological principles relative to technology design, (2) a stakeholder engagement process that incorporates principles of patient-centered care, and (3) content development and strategies to reinforce learning. The subsequent RCT [19] will test the effect of the mobile technology developed by our team on transition readiness as a primary end point.

## Methods

We developed the MyREADY Transition BBD app as a stage of the READYorNot BBD trial (RCT; ClinicalTrials.gov NCT03852550) [19].

### Patient Population

This project was conducted within the Child Health Initiatives Limiting Disability–Brain Research Improving Growth and Health Trajectories (CHILD-BRIGHT) pan-Canadian network. The CHILD-BRIGHT projects focus on optimizing the health and well-being of infants, children, and youth with brain-based developmental disabilities [20]. Eligibility for enrollment in the READYorNot BBDs RCT [19] included patients who are aged between 15 and 17 years; have a diagnosis of autism spectrum disorder, cerebral palsy, epilepsy, fetal alcohol spectrum disorder, or spina bifida; have cognitive ability to provide informed consent; have the ability to read and understand English or French; have access to the internet and a smartphone, iPad, tablet, or desktop computer; and have a TRANSITION-Q score of >40 to benchmark the minimum threshold for transition readiness based on our earlier work [21]. In our app development, we decided on a target population of BBDs, including conditions that originate before an individual reaches the age of 18 years; that continue or can be expected to continue indefinitely; and that constitute a substantial impairment in three or more areas of major life activity including (1) self-care, (2) receptive and expressive language, (3) learning, (4) mobility, (5) self-direction, (6) capacity for independent living, and (7) economic self-sufficiency. In addition, we considered the most prevalent BBDs among adolescents who were followed in the Canadian recruitment sites who would participate in our subsequent trial designed to test the efficacy of the mobile app. For the trial, we developed a detailed diagnostic reference document for recruitment; for example, adolescents with spina bifida occulta without any neurological loss and spina bifida of any type that has not resulted in hydrocephalus and developmental disability were excluded. The rationale for targeting individuals aged 15 to 17 years stems from several key considerations. This age group represents a cohort that is typically approaching the transition from pediatric to adult health care services, making them particularly relevant for the intervention’s focus on transition support. By targeting adolescents who are nearing the transition period, we can ensure that they have ample opportunity to engage with the intervention and benefit from the support provided by the app. By selecting adolescents who are close to transition but still within a developmental stage conducive to learning and adaptation, we aim to maximize the effectiveness and impact of the intervention on their transition readiness and outcomes.

### Methodological Principles Relative to Technology Design and Development

We assembled a multidisciplinary team of technology developers (HIT platform design); transition psychologists (content development); and stakeholders (patients, family members, and clinicians). The HIT team (AM, RR, and AV-DL) included technology experts, patient-centered care experts, health services researchers, and industry partners. We partnered with 360Medlink, a software company with experience in the development of multimedia solutions in the health care industry [22]. The transition psychology and informational content team (AHK, KA, CBF, BG, AV-DL, AM, and JWG) included clinical psychologists and transition experts in BBD who led the development of the educational content. The stakeholder engagement team (JWG, RR, SS, BG, AM, and AV-DL) included experts in developmental pediatrics and neurology and patient engagement to lead the stakeholder engagement activities and reports. Detailed information on the expertise and demographics of the teams is shown in Table 1.

**Figure 1:**
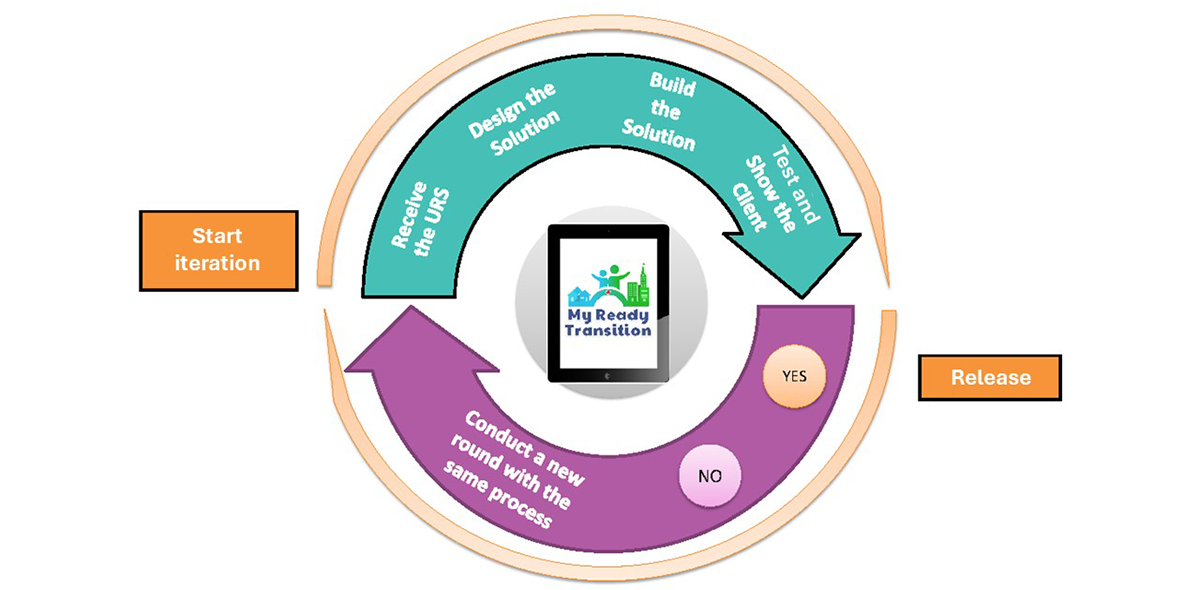
Description of agile iterative methodology. The figure provides a visual illustration of the agile iterative methodology applied to the life cycle of concept, app, and content development. URS: user requirement specification.

**Figure 2:**
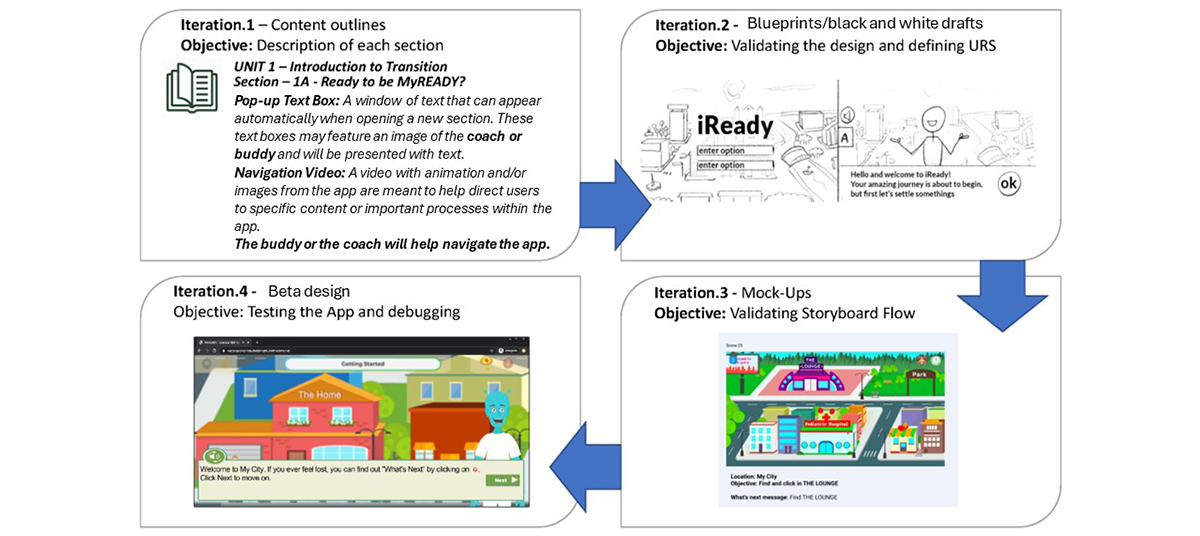
Interface development showing progress from descriptions to wireframes to final version. The figure illustrates the interface developmental process. All the design elements (buildings, rooms, objects, mentor, and accessories); logo; and the content (mentor messages, videos, and challenges) followed this developmental 4-step process. URS: user requirement specification.

To maintain continuous responsivity to feedback from stakeholders, we adhered to principles of Agile software development [23,24]. This programming approach enables dynamic collaboration with users as illustrated in Figure 1. The 3 teams communicated weekly to make iterative adjustments to the app based on feedback. We tested proposed solutions in subsequent consultations and repeated this process until a final solution was reached. An example of a storyboard reiteration is shown in Figure 2.

**Table 1:** Description of the multidisciplinary teams.

Team					Values
**Health information technology (n=7)**					
	**Age (y), range**				
		Technology project manager	40-55		
		Health services researchers	45-60		
		Industry partner team	25-50		
	**Sex, n (%)**				
		Female	2 (29)		
		Male	5 (71)		
	**Expertise, n (%)**				
		Technology project manager	1 (14)		
		Health services researchers	2 (29)		
		Industry partner team	4 (57)		
**Psychology content development (n=9)**					
	**Age (y), range**				
		Lead clinical psychologists	40-60		
		Trainees	25-35		
	**Sex, n (%)**				
		Female	7 (78)		
		Male	2 (22)		
	**Expertise, n (%)**				
		Lead clinical psychologists	2 (22)		
		Project managers	2 (22)		
		Health services researchers	2 (22)		
		Trainees	3 (33)		
**Stakeholder engagement (n=6)**					
	Age (y), range			25-55	
	**Sex, n (%)**				
		Female	4 (67)		
		Male	2 (33)		
	**Expertise, n (%)**				
		Experts in developmental pediatrics and neurology and health services researchers	2 (33)		
		Patient-centered care expert	1 (17)		
		Qualitative data analyst	1 (17)		
		Project managers	2 (33)		
**Patient and family advisory council (n=9)**					
	Age (y), range			17-60	
	**Sex, n (%)**				
		Female	5 (56)		
		Male	4 (44)		
	**Expertise, n (%)**				
		Adolescents in transition	1 (11)		
		Young adults at posttransition stage	3 (33)		
		Parents	5 (56)		

### Stakeholder Engagement Process

**Figure 3:**
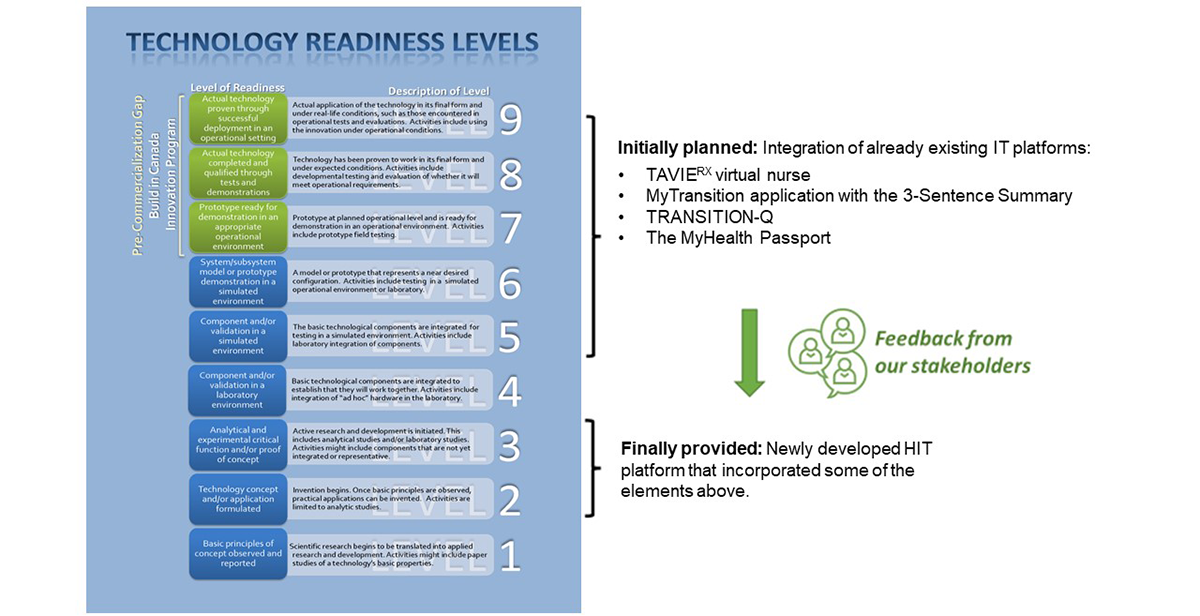
Description of technology readiness levels. The figure defines technology readiness levels used to predict the process from innovation concept to commercialization. HIT: health information technology. The left panel of the figure (Technology Readiness Levels) is a copy of the version available at https://buyandsell.gc.ca/initiatives-and-programs/build-in-canada-innovation-program-bcip/program-specifics/technology-readiness-levels.

App development was a collaborative process involving a series of research and stakeholder engagement activities. We used a participatory approach that incorporated stakeholder engagement and created an app that was patient centered and aimed for the highest probability of effectively meeting the needs of the BBD population. Our approach to eliciting feedback and making decisions was grounded in the principles of collaboration, transparency, and pragmatism to ensure that stakeholder input was carefully considered and incorporated wherever feasible [25]. We hypothesized that elements of existing patient-facing HIT tools could be leveraged to create a novel population-specific app. We reviewed patient-facing apps that could be adapted to meet our needs, including the TAVIE virtual nurse platform designed to improve patients’ health knowledge and self-efficacy through guided need-specific content [26]. We evaluated existing BBD transition tools, including the MyTransition app [27], which includes resources to support health care transition including a 3-sentence summary to teach adolescents a concise method of summarizing their medical condition [28]; TRANSITION-Q, a validated scale for measuring skills needed to manage one’s own health care [21]; and the MyHealth Passport, a web-based tool developed by the Good2Go Transition Program [29] of the Hospital for Sick Children in Toronto [30]. We hypothesized that we could incorporate existing tools for the app development based on a technology readiness level between 5 and 6 of 9 levels, a method used to assess the maturity of technology relative to acquisition readiness [31] (Figure 3). We planned a 12-month pre-RCT development phase aiming to achieve a technology readiness level of 8, meaning that the app technology has been proven to work in its final form and under expected conditions and is now ready to be deployed and tested in real-life situations [31].

We adopted a patient-oriented research methodology, engaging key stakeholders (youths with disabilities, families, and clinicians) and integrating their input throughout the design and development process. We hosted a stakeholder knowledge exchange activity to interact with existing HIT tools and discuss priorities before beginning app development. We formed a patient and family advisory council (PFAC) comprising a group of stakeholder partners including 1 adolescent in transition, 3 young adults at posttransition stage, and 5 parents who partnered with us on key discussions and decision-making. Among the adolescents and young adults, 2 were male and 2 were female. These youth and parent partners had a collective lived experience of cerebral palsy, autism spectrum disorder, stroke with hemiplegia, mental health conditions, prematurity with chronic health conditions, rare disorders, and complex medical needs. We hosted regular meetings with the PFAC to garner input about the concept and discuss the contents of the app. We conducted a focus group to help inform and finalize the proof-of-concept beta version of the app. We observed and interviewed adolescents and young adults and parents during an app beta version test. Adolescents, young adults, parents, and clinicians later participated in app usability testing by downloading and installing the app, testing it for 10 days, completing a worksheet and questionnaire, and sharing their experience during a qualitative interview to field-test the acceptability of the tool principally at the patient and family level and point of care to adjust and optimize the intervention tool before the subsequent clinical trial phase.

**Figure 4:**
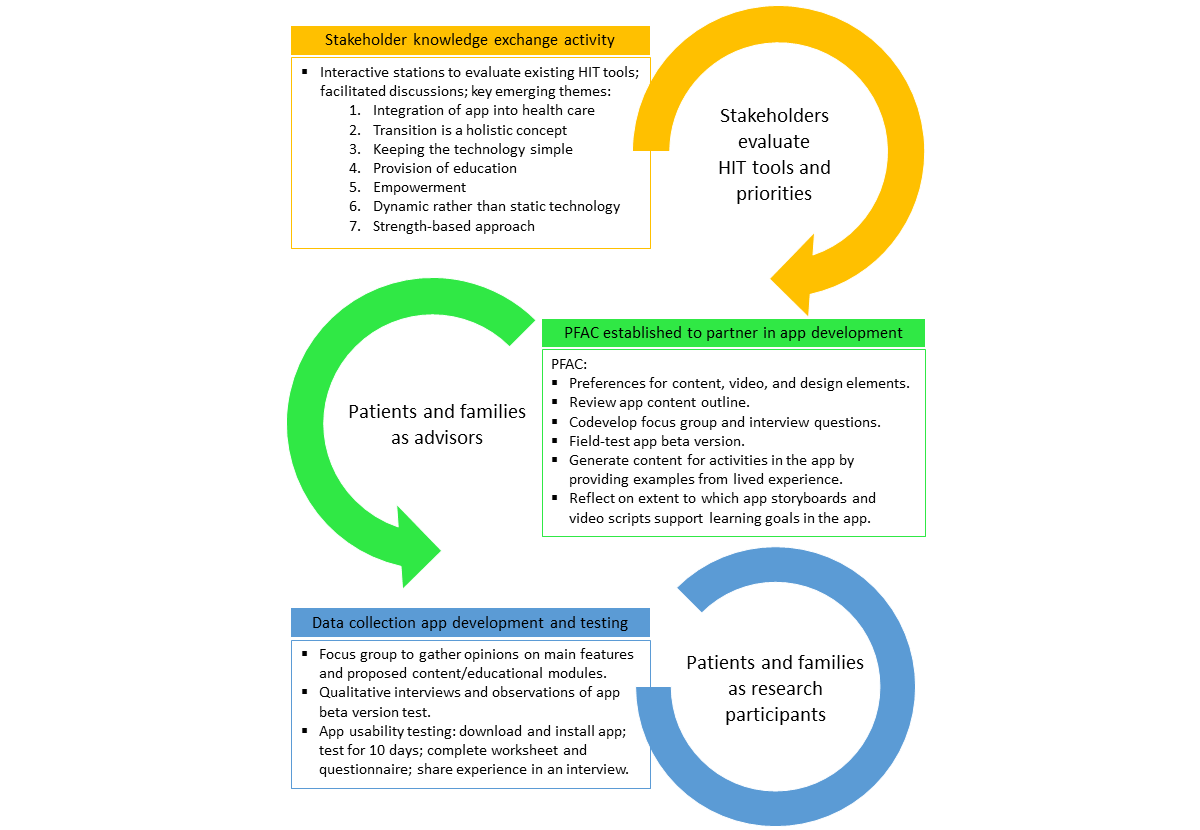
Illustration of the stakeholder engagement process and activities. HIT: health information technology; PFAC: patient and family advisory council.

The user requirement specification list was created collaboratively by the HIT team described in Table 1. This collaborative effort drew upon extensive experience in similar health care and pharmaceutical projects, as well as adherence to best practices in app development within the health care sector. The list captured the functional and nonfunctional requirements of the app, thereby guiding its development and ensuring its alignment with stakeholder needs and regulatory standards (details in Multimedia Appendix 1). To finalize the inclusion of the user requirements, after consultation with the PFAC, 4 members from each of the psychology content development team and the stakeholder engagement team were asked to adjudicate the final inclusion or exclusion of the technical specification. The list was shared among all the stakeholders to define the minimum viable product for the app [32,33]. The process of stakeholder engagement is illustrated in Figure 4 and described in Multimedia Appendix 2.

### Content Development

The transition psychology and informational content team designed preliminary content tailored to the specific needs of the BBD population. In alignment with what we learned from our research and stakeholder engagement activities, the goals were education and self-management through guided activities [15,34]. We tailored the content directly to youth while remaining mindful of the needs and expectations of other stakeholders including parents or guardians and health care providers [16].

Grounded in the principle of cognitive learning theory [35], we framed the learner as an active participant engaging with information from internal and external sources. Information was presented via a gamified interface in which a user navigates through a learning environment with the support of a guide and visits various areas of the learning environment to move through the content at a self-directed pace; this fosters motivation and intervention adherence while moving through educational sessions and achieving specific learning objectives.

The content and format of the chapters were developed systematically based on the core values and principles that emerged from stakeholder consultations and subjected to the user requirement specification list and minimum viable product criteria. Principles of social learning theory emphasized environmental and cognitive factors as well as the importance of modeling positive behavior, informed the development of a visual depiction of a city that the user learns to navigate, and included a mentor to guide users through the content. We incorporated quizzes, mentor dialogue, educational videos, as well as challenges and activities during each session. We created opportunities for self-evaluation, including a visual progress bar, a sliding scale for self-assessment of transition readiness, and checkpoints. We included a toolbox of transition resources for users, enabling access to key information about their health condition and treatment plan, thereby facilitating patient-provider communication. We cocreated multimedia content working with Spectrum Productions [36], a nonprofit social enterprise employing individuals on the autism spectrum, to film conceptual and testimonial videos with patient-actors.

### Ethical Considerations

Hamilton Integrated Research Ethics Board (HiREB 2952) approval was obtained. For components of the study where patients and families were engaged as research participants (focus group, formative usability test, and summative usability test), adolescent participants provided assent and parents or caregivers provided consent for the participation of their child and themselves. We adhered to local, national, regional, and international laws and regulations regarding protection of personal information, privacy, and human rights.

## Results

### Technology Readiness Level and Elements

The app was developed from June 2017 to June 2019, and the English language version was launched in iOS and Android versions in the App Store and Google Play, respectively, on June 19, 2019. This was 12 months longer than (ie, twice as long as) the anticipated time in the funded grant application. Although the resources used in our preliminary design served as a starting point for development, feedback from our stakeholders motivated the creation of a completely newly developed HIT platform, reducing the technology readiness level from 5 to 6 at the onset of our study to a level of 2 to 3 as characterized in Figure 3.

The READYorNot BBD intervention includes 3 elements: the MyREADY Transition BBD mobile app, a content library containing the transition curriculum, and a back-end platform that manages the data and features of the user interface. To minimize time requirements and costs, the app was developed in Unity, a cross-platform tool, that works primarily with iOS and Android devices as well as with internet browsers. The final app comprises 985 storyboards or scenes; 88 testimonial and 46 conceptual videos; and 47 reward-driven challenges in English and in French.

Content management capabilities include indexing, search and retrieval (search boxes on the forms and reports of the indexed data by attributes such as publication dates, keywords, or population); content hierarchy with unlimited depth and size and an integrated file manager for video with multiple language support; format management (ensuring that content and videos fit the format and structure required for download); revision control (enabling content version management, including duplication and edition after initial publication); and publishing (which pushes the content in a digestible and preformatted way via the app user interface).

On the back-end platform, the app is run by a content management system that administers the users’ information, journey completion (progression and adherence), and perceived value data. The content management system was designed using open-source resources, including the MySQL relational database, allowing for greater flexibility and customization as well as more timely development and scalability, while minimizing the costs associated with using branded solutions [37]. The content management system automatically pushes notifications to users based on predefined content deployment rules as well as user activity and enables administrators to access a dashboard containing user analytics reports. The content management system supports several administrative features in an easy-to-use interface, including onboarding, offboarding, secure authentication of users’ access, monitoring users’ data and activity, and push notifications to enhance users’ adherence.

The dashboard (initially supported by Sisense data analytics software [Sisense] and currently by Microsoft Power-BI [Microsoft Corp]) contains user demographics, device type, operating system, user progression, and perceived value as well as options for data exportation. The dashboard incorporates preconfigured graphs and reports that can be filtered by key performance indicators.

The app requires an internet connection during the initial download and setup and for certain features (eg, video playback and voiceover). To ensure that users maintain access to the content, we designed the app to function offline if necessary. To do so, we optimized the balance between maximizing offline availability of key features and minimizing the download size.

### Customization and Personalization of the App Based on Stakeholder Engagement

Key emerging themes from the stakeholder engagement process described in Figure 4 included the importance of viewing transition as a holistic concept, the need to keep the technology simple, and the central role of education. To increase the patient-centered focus of the app, in response to the PFAC, we incorporated features that were customized to the population with BBDs as well as options to personalize the experience of using of the app based on preferences and needs. True to the process of engagement, we also incorporated features to maximize adherence. The key features that were enhanced through customization or personalization are summarized in Multimedia Appendix 3. Features that can be both customized and personalized include privacy settings and notification, mentor appearance, text-to-speech and volume control, choice of language, color contrast and animation, and tailored resources.

### Content Output

**Figure 5:**
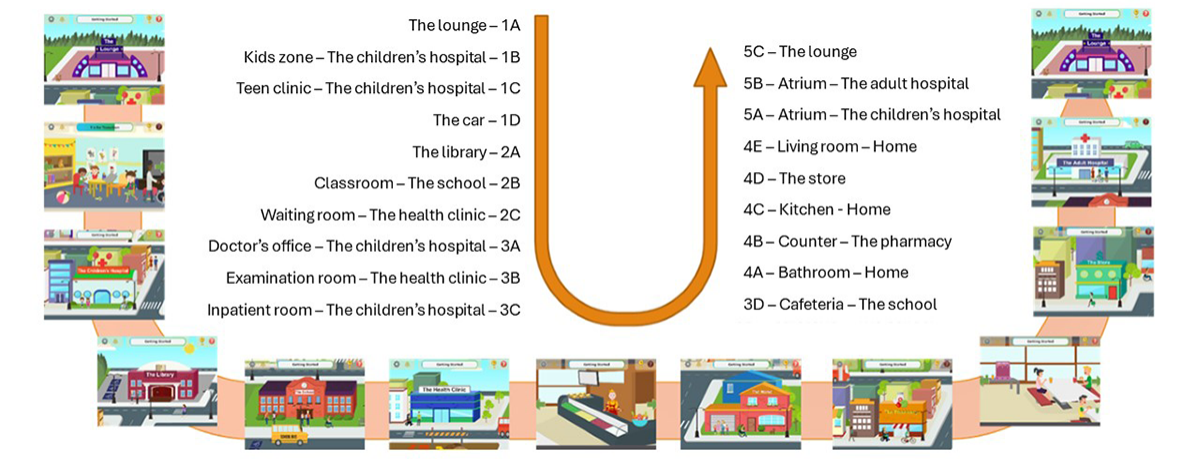
Content scheme and session environment. Content scheme and session environment as user advances through the “Journey in the City,” an internet-based environment that reinforces learning through gamification. In the app, the user navigates through a simulated city (“My City”) by engaging with a series of internet-based buildings, each containing a thematic educational session. The user’s navigation is based in "the lounge," which acts as the starting point for each session.

The content is deployed in five educational chapters: (1) Introduction to Transition; (2) Knowledge is Power; (3) Communication is Key; (4) Time to Take Charge; and (5) The Other Side (ie, adult care). Up to 19 sequential educational sessions are distributed along the chapters as illustrated in Figure 5. The subdivision of the educational curriculum into multiple sessions ensures that the content delivery is optimized to the attention span of youth with BBD. Each session contains between 40 and 55 scenes and includes an average of 7 videos covering conceptual material and testimonials from adolescents with BBD. The learning environment is presented in the context of a “Journey in the City,” an internet-based environment that reinforces learning through gamification.

**Figure 6:**
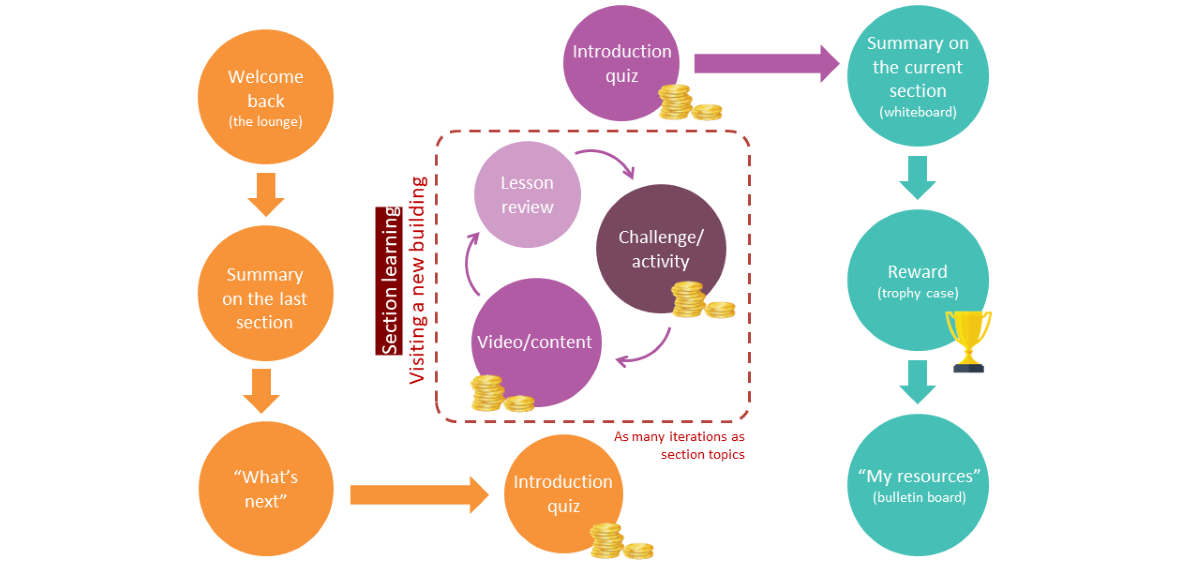
Workflow and recursive structure of educational sessions. The sessions have a recursive structure, and their predicable nature is intended to facilitate navigation through the content. Each session begins at the same location and guides the user to a new location to access the main content and activity of that session. The sessions conclude by guiding the user back to the start location while providing a summary of the key concepts learned and an opportunity to collect a reward.

The transition curriculum is delivered through a gamified user experience in which the content of each session is provided through a combination of activities including challenges, games, and quizzes. These activities are designed to maintain the user’s interest by introducing problem-solving elements and allowing users to collect rewards upon completion. The sessions have a recursive structure, and their predictable nature allows the user to easily navigate through the content. Each session begins at the same location and guides the user to a new location to access the main content and activity of that session as illustrated in Figure 6. The sessions conclude by guiding the user back to the start location while providing a summary of the key concepts learned and an opportunity to collect a reward.

Throughout their journey, the user is accompanied by a mentor that guides them through the learning environment, progressively introducing each educational activity, delivering informational messages, reminders, and motivational reinforcements. The user can build a relationship with the mentor, which simulates contact with a peer who has been through the transition process and facilitates learning by modeling positive behavior [3,38]. Users can customize the mentor’s appearance as they accumulate rewards by completing activities.

## Discussion

### Principal Findings

In this study, we describe our experience developing a novel digital platform (MyREADY Transition BBD app) that delivers educational content through an internet-based gamified interface designed to engage and empower adolescents with BBD to navigate health care transition. To our knowledge, this is the first HIT intervention intended to improve transition readiness for youth with BBD. We hypothesized that existing tools could be migrated into the app to address education, empowerment, and navigation. Stakeholder feedback led us to create a custom-designed platform. Furthermore, although the development of the transition curriculum incorporated principles of cognitive and social learning theory, we found that the educational content needed to be delivered in a way that captivated the users. This led us to adopt gaming features and internet-based multimedia expression of contents to harness learning styles of youth with BBD. Finally, perhaps the most challenging but also rewarding aspect of our iterative design process was the rigorous incorporation of stakeholder engagement, requiring a back-end technology management platform that was cumbersome and required labor-intensive hands-on support. Our experience provides insight for other app developers, health care providers, and researchers about the process and efforts needed to develop apps for youth with special needs.

Youth with lifelong health conditions exhibit substantial variability in transition readiness [11-13]. Since policy-driven transfer of care is variable, we sought to develop an intervention that improves transition readiness. There is an increasing need to support the process of health care transition in a way that is adaptive to the demands of the adult health care system [4]. As an increasing number of HIT platforms designed to meet care gaps for specific populations are being developed in partnership with patients and industry, adjustment of time, budget, and expectations need to be carefully managed to achieve timely delivery of high-quality end products. The gaming elements, multimedia features, and journey-like processes throughout the app were designed to foster engagement and adherence to the educational curriculum [39]. Throughout the content development, stakeholders highlighted the need to deliver the transition curriculum using a strategy that is adapted to the learning requirements of youth with BBD [12]. This required a creative approach that merges the principles of cognitive and social learning theory with contemporary gamification strategies [40] and evidence on health intervention technology preferences among youth [12,41,42]. We incorporated several gaming elements into the app, including an internet-based environment, objective-based learning modules, challenges, and rewards [39]. In tandem with this, the mentor simulates peer-supported social learning, which has been identified as an important facilitator for learning among youth with BBD [3,38]. Gamification has the potential to reinforce learning and increase engagement with the transition curriculum [43-45].

Patient-centered, patient-facing mobile apps offer a promising approach to supporting health care transition by educating, engaging, and empowering youth and their families to manage their own health in collaboration with their health care providers [18]. Our project aligns with translational research design methods where the philosophy for using patient-centered design is to understand the needs of the people at the center of the research. Designing research in this way, based on an understanding of the needs and knowledge of user-centered design principles, aims to reduce the gap between research and its application. Our efforts to understand the users at the center of the project are evident both in our research approach and in our iterative methods to develop the app based on stakeholder feedback. Gelinas et al [46] provided recommendations from a Delphi panel regarding the oversight of patient-centered outcomes research, including the recommendation to use a formal taxonomy of patient involvement in research to disambiguate and distinguish between 3 broad roles patients may take, namely, study personnel, advisor, and research participant. In our project, we engaged patient and family stakeholders as advisory members in some instances and as research participants in other instances. We used these complementary approaches to garner input during the app development and usability testing process and to embed patients and families as formal advisors in key discussions and decision-making. We applied this user-centered, participatory approach by design to create a patient-centered app with a high probability of effectively meeting the needs of the population with BBDs. Many of the content and design decisions were made in collaboration with the READYorNot PFAC. The PFAC for this project was diverse and included both caregivers and youth in various stages of transition, some of whom transitioned to adult care during their time on the project. Having input from those currently living the transition experience allowed the app to be shaped in real time by youth’s changing perspectives. While these partnerships were invaluable to the success of our app development, it is understandably difficult to recruit and retain youth to be involved in research; it requires time and flexibility in order to build a working relationship that may not be feasible in some circumstances. Researchers who are finding it challenging to engage youth may choose a consultation service such as the Child and Youth Advisory Council through Alberta Health Services or the National Youth Advisory Panel within the CHILD-BRIGHT network. Use of consultation services can be complementary to the other partnership activities, as there is often a need to use a suite of engagement tools when engaging youth with busy schedules.

We used an iterative technology methodology to incorporate stakeholders’ feedback. It was difficult to adapt existing tools due to our commitment to personalization and customization of app features guided by our PFAC. Our iterative design required time-consuming back-end technology management. Developing the platform took 24 months instead of our grant-approved 12 months, impacting the planned trial onset within the allotted budget. An increasing number of HIT tools are being developed to meet care gaps [42,47,48]. Many of these tools are designed for patients with specific health conditions but may not be tailored to the needs and characteristics of specific populations, which can negatively impact adherence [49]. We aimed to design a patient-centered HIT intervention for youth with BBD, a population with a range of specific health conditions as well as specific usability needs, which posed unique design challenges [50,51]. Although numerous public funding agencies are calling for patient partners in research design and implementation, guidelines that estimate the impact of this engagement on achieving study deliverables are scant. Moreover, industry partners, pivotal to the success of product development, are to date, ill-equipped to handle the excess labor required to achieve this. Thus, despite the potential benefits of stakeholder engagement, the implementation of these partnerships remains challenging for HIT tool development, as it involves additional logistical considerations, significantly more time and funding, and careful management of stakeholders’ expectations and responsibilities. As such, the co-design process requires a shift away from traditional health research processes and timelines.

In our study, we define *education* as the provision of information and resources aimed at enhancing participants’ understanding of various aspects related to their health care and transition process. This includes imparting knowledge about their medical condition and health care rights and responsibilities, as well as empowering them with skills to effectively advocate for their needs within the health care system. Empowerment refers to the process of equipping individuals with the confidence, skills, and self-efficacy to actively participate in decisions regarding their health and health care. This involves fostering a sense of autonomy, self-advocacy, and self-management, empowering participants to take ownership of their health and well-being. Health system navigation encompasses the ability to navigate and access health care services effectively in Canada, including understanding how the health care system operates, accessing appropriate services and resources, and overcoming the barriers to care. These domains were selected based on their significance in facilitating successful transitions to adult health care and improving health outcomes among adolescents with chronic conditions.

### Limitations

We achieved our goal although the time required to create the app exceeded the time that we planned initially. We chose to design a mobile app to be used by youth rather than parents or clinicians. While the latter are also important stakeholders in supporting health care transitions, youth are at the forefront of this process and stand to benefit the most from this type of intervention. With respect to the design process, several features that were suggested during the stakeholder consultations were omitted from the app due to budget, feasibility, and time constraints, leading to challenges with balancing the needs and expectations of stakeholders against the complexity of the resulting platform. A related challenge was to manage the contrasting timelines of the overarching research project and the software development. Finally, the effectiveness of this app in improving transition outcomes is yet to be evaluated. We did not systematically measure stakeholder engagement and satisfaction with our design approach of partnering with patients and industry to develop the app. However, we have published a commentary on navigating meaningful engagement and lessons learned from partnering with youth and families [52].

We recognize it is a limitation that the app is accessible only to people with a smart device or a computer with a navigator and internet access. While we have designed an offline mode, an internet connection is still required for initial download of the app, and a continuous internet connection is essential for accessing videos and text-to-speech features and synchronization with the content management system. Once we have completed the RCT and determined which components of the intervention have the highest probability of improving transition readiness, we will incorporate those in the future iterations of the intervention and explore how to maximize the accessibility of the app. Moreover, future iterations of the platform may incorporate measures to mitigate disparities in access to technology and internet services and digital literacy, thus aligning with broader goals of promoting health equity. Despite the limitation, mobile apps are a complementary approach to reducing care gaps, addressing health inequities by improving access to resources and support that may not be available in the clinical environment.

The importance of experimental implementation cannot be overstated. Although implementation science has made great advances in the use of digital technologies to advance knowledge translation and improve self-management and adherence, rigorous clinical trials need to measure their impact on well-specified outcomes. The process of engagement of patients as partners in research needs to be well characterized and accounted for in research planning. The MyREADY Transition BBD app will be implemented and tested in the READYorNot BBD trial, an ongoing cluster RCT, which began recruitment in June 2020 in 4 regions across Canada [19]. The objective of this trial is to assess the impact of this innovative tool on transition readiness among youth with BBD. The trial is designed to include 264 adolescents aged 15 to 17 years with BBD who meet minimum health and technology literacy criteria in order to potentially benefit from this intervention. We will collect and analyze data pertaining to the usability and patient experience of the app as well as how the app operates to further optimize the intervention. We hope that this intervention will be used by youth with BBD and be endorsed by health care providers and experts in Canada and elsewhere.

### Conclusions and Future Directions

The MyREADY Transition BBD mobile app delivers educational content through an internet-based gamified interface, which was designed to engage and empower youth with BBD to navigate health care transition. The impact of this app on transition readiness and outcomes is being measured with an ongoing trial, the results of which will guide future development of apps for youth with special needs and steps toward dissemination. This intervention has the potential to improve transition outcomes during health care transition from pediatric to adult health care systems. Our process has the potential to inform other researchers tackling the growing need for HIT tools to support patients and families transitioning through the health care system. Technology platforms developed in partnership with patients and industry can be patient centered, but careful resource management is needed to achieve timely delivery of end products for the much-needed trials that will inform their application in clinical practice.

## Appendix

Multimedia Appendix 1 User requirement specification creation.

Multimedia Appendix 2 Description of the process of stakeholder engagement.

Multimedia Appendix 3 Description of MyREADY Transition BBD app’s key customization and personalization features.

## Abbreviations

BBD: brain-based disability
CHILD-BRIGHT: Child Health Initiatives Limiting Disability–Brain Research Improving Growth and Health Trajectories
HIT: health information technology
PFAC: patient and family advisory council
RCT: randomized controlled trial
READYorNot: Readiness in Youth for Transition Out of Pediatric Care
