# Supplementary material for: Development of MyREADY Transition BBD Mobile App, a Health Intervention Technology Platform, to Improve Care Transition for Youth With Brain-Based Disabilities: User-Centered Design Approach
Source: JMIR Pediatr Parent. 2024 Oct 1;7:e51606. doi: 10.2196/51606 (PMC11480690; doi:10.2196/51606)
Supplement: Multimedia Appendix 1 [file pediatrics_v7i1e51606_app1.docx]

User Requirement Specification creation

To create a comprehensive User Requirement Specification, the HIT team presented a list of requirements and features to the multidisciplinary teams for their evaluation. Careful consideration was given to each one, with the team assigning varying weights based on their relevant expertise.

The creation of such specifications often aligns with regulatory standards such as those outlined by Health Canada's Medical Devices Regulations. These regulations provide guidelines and requirements for the development and deployment of medical software applications to ensure their safety, efficacy, and adherence to quality standards. Additionally, other regulatory bodies such as the Canadian Agency for Drugs and Technologies in Health or The Personal Information Protection and Electronic Documents Act may provide further guidance on the development and implementation of healthcare technologies.

Finally, research on diverse IT publications as well as Gartner's reports and insights on digital health trends, technology adoption, and best practices were known by the HIT team and showed to be valuable resources for organizations developing healthcare software applications, as they offer industry perspectives and recommendations based on market research and analysis.

Supplement table. Summary of questions used to inform final user requirement specifications:

| ***line*** | **Questions** | **Final inclusion** | **Response** | | |
| --- | --- | --- | --- | --- | --- |
|  |  |  | **N = YES** | **N = NO** | **N = MAYBE** |
|  | **MENUS** |  |  |  |  |
| *1* | Do we want to have a Top Left Menu? | Yes | 6 | 1 | 1 |
| *2* | Are the options for the Top Left Menu correct? | Yes | 4 | 2 | 2 |
| *3* | Would you add something? *If yes, please detail.* | Yes | 4 | 4 | 0 |
| *4* | Do we want to have a Bottom Bar with buttons? | Yes | 8 | 0 | 0 |
| *5* | Are the options for the Bottom Bar correct? | Yes | 5 | 2 | 1 |
| *6* | Would you add something? *If yes, please detail.* | Yes | 4 | 3 | 1 |
|  | **THE PATIENT PROFILE** |  |  |  |  |
| *7* | Do we allow the user to incorporate his name? | Yes | 8 | 0 | 0 |
| *8* | Do we use a nickname instead? | Yes | 5 | 3 | 0 |
| *9* | Do we incorporate more detailed information? *Open information such as description, history, etc.* | Yes | 6 | 2 | 0 |
| *10* | If so, it can be filled up as part of his registration process *(and completed with the help of healthcare professionals or parents)* | Yes | 6 | 0 | 2 |
| *11* | Do we make this information exportable? *e.g., eMail.* | Yes | 7 | 1 | 0 |
| *12* | Do we allow the user to incorporate his photo? | Yes | 4 | 3 | 1 |
| *13* | Do we use an avatar of user’s choice instead? | Yes | 7 | 0 | 1 |
| *14* | Do we allow the user to change the information anytime without parents of healthcare professionals supervision? | Yes | 6 | 1 | 1 |
| *15* | Do we add a list of contacts? *Emergency or not.* | Yes | 7 | 1 | 0 |
|  | **ABOUT THE JOURNEY (OR THE MAZE)** |  |  |  |  |
| *16* | Do we use the Journey (or Maze) as a concept? | Yes | 2 | 2 | 4 |
| *17* | Do we use the Journey to evaluate the progression of the patient? | Yes | 6 | 0 | 2 |
| *18* | Shall the user go over the Journey only one time? | No | 1 | 6 | 1 |
| *19* | If he can do the Journey several times, can the user change his results once completed? | Yes | 6 | 0 | 2 |
| *20* | In case the user can do the Journey more than one time, do we store the results to be compared in the future? | Yes | 6 | 0 | 2 |
|  | **MODULES AND SESSIONS** |  |  |  |  |
| *21* | Can the user access to more than one module simultaneously? *e.g. The Disease + Appointments* | Yes | 4 | 4 | 0 |
| *22* | Will the user profile (his personal and medical information) pre-define a list of specific modules? *If not he will see all the same modules as all the rest of patients on regards if they share or not the same medical condition.* | Yes | 6 | 2 | 0 |
| *23* | Will the sessions be composed of only video or PDF? | No | 3 | 4 | 1 |
| *24* | Will the videos be person? | Yes | 8 | 0 | 0 |
| *25* | Will the videos be an avatar? *If so we may have limited ones and not be editable for the user.* | No | 1 | 4 | 3 |
|  | **THE SESSIONS** |  |  |  |  |
| *26* | Would we make a question after a session to assess his knowledge? | Yes | 8 | 0 | 0 |
| *27* | Do we want to track this information? *(Session completion).* | Yes | 8 | 0 | 0 |
| *28* | Do we want the parents to be aware of the evolution? | No | 2 | 4 | 2 |
| *29* | Do we want the healthcare professionals to be aware of the evolution? | No | 3 | 4 | 1 |
| *30* | Can the user access to one session before completing the previous one? | No | 3 | 5 | 0 |
| *31* | Can the user view once again a session already completed? | Yes | 8 | 0 | 0 |
| *32* | Can a session be available depending of the answer for a previous question? *e.g. If the user answer NO for the question for module1 session1, then the module5 and session5 will be unblocked?* | Yes | 6 | 1 | 1 |
| *33* | May we reward (points) to the patient if he/she is completing the sessions? | Yes | 5 | 2 | 1 |
|  | **THE QUESTIONS** |  |  |  |  |
| *34* | Do we want to track this information? | Yes | 8 |  |  |
| *35* | Do we want the parents to be aware of the results? | No | 1 | 6 | 1 |
| *36* | Do we want the healthcare professionals to be aware of the results? | No | 1 | 6 | 1 |
| *37* | Can the user go back to change his response to a question any time? | Yes | 6 | 0 | 2 |
| *38* | If the user can re-do the questions, do we compare first results vs. next results? *(As an evolution)* | Yes | 6 | 0 | 2 |
| *39* | If the user can re-do the questions, how many times it will be available? *(Consider the trial stats)* | Yes | 5 | 0 | 3 |
| *40* | May we reward (points) the patient if he/she is completing the questionnaires? | Yes | 5 | 2 | 1 |
|  | **THE TASKS - AGENDA** |  |  |  |  |
| *41* | Do we want to track this information? | Yes | 6 | 0 | 2 |
| *42* | Do we want the parents to be aware of? | No | 3 | 3 | 2 |
| *43* | Do we want the healthcare professionals to be aware of? | No | 1 | 5 | 2 |
| *44* | Do we want to send reminders to the user? | Yes | 6 | 0 | 2 |
|  | **SETTING UP A TASKS OR APPOINTMENT** |  |  |  |  |
| *45* | Would tasks or calendar be useful for the patient? | Yes | 6 | 0 | 2 |
| *46* | Shall we add more information? *e.g. Address, access maps, etc. for an appointment.* | Yes | 3 | 2 | 3 |
| *47* | May we reward (points) him if he is completing tasks? | Yes | 6 | 2 | 0 |
| *48* | Do you think there should be any other type of tasks than medical appointment? *E.g. Medication?* | Yes | 8 | 0 | 0 |
|  | **THE PROGRESS** |  |  |  |  |
| *49* | Do we want to user to see graphically how they are performing? | Yes | 8 | 0 | 0 |
| *50* | Do we give them feedback vs. other patients’ average? | No | 0 | 8 | 0 |
| *51* | Do we want the parents to be aware of? | No | 1 | 5 | 2 |
| *52* | Do we want the healthcare professionals to be aware of? | No | 2 | 5 | 1 |
|  | **ABOUT THE TRAQ or Transition Q** |  |  |  |  |
| *53* | Do we use the TRAQ (or Transition Q) to evaluate the progression of the patient? | Yes | 6 | 1 | 1 |
| *54* | Will the user go over the TRAQ only one time? | No | 2 | 6 | 0 |
| *55* | Can the user change his results once completed? | No | 3 | 4 | 1 |
| *56* | Would it be accessible throughout the Journey? *If not it may be throughout his profile.* | Yes | 4 | 0 | 4 |
| *57* | Will the user answer the TRAQ before the Journey? *e.g. First step of the Journey.* | Yes | 7 | 1 | 0 |
| *58* | Will the user answer the TRAQ after the Journey? *e.g. Last step of the Journey.* | Yes | 7 | 0 | 1 |
| *59* | In case he completes more than one time, will the user be congratulated if he performed better on the TRAQ? | Yes | 5 | 2 | 1 |
|  | **General Questions 1/2** |  | | **ANSWER** | |
| *60* | Which platform we develop the App first? iPhone Operating System *(iPhone / iPad) or Android?* |  | | Version 1 iPhone Operating System / Version 3 (CT) both | |
| *61* | Do we develop the patients’ application to work desktop instead of for a mobile device? |  | | Version 1 No / Version 3 (CT) Yes | |
| *62* | Do we track the use of the application? *e.g. Click on buttons.* |  | | Yes | |
| *63* | How much time we want the user to engage to the application (weeks, months…)? |  | | 8-16 weeks | |
| *64* | How much time do we expect the user to invest on the application daily? |  | | 1-2 times a weeks | |
|  | **General Questions 2/2** |  |  |  |  |
| *65* | Shall we consider to incorporate a community tool between patients? *e.g. Forum, members chat. If so, we need to incorporate a moderator?* | No | 3 | 4 | 1 |
| *66* | Shall we consider to incorporate a communication tool patients- healthcare professionals? *e.g., 1:1 chat or email* | No | 0 | 8 | 0 |
| *67* | Shall we consider to incorporate a communication tool between patients? *e.g., 1:1 chat or email* | No | 2 | 6 | 0 |
| *68* | Shall we use AI (Artificial Intelligence) to be able to answer open questions from patients such as IBM-Dr. Watson? *If so, we will define the sources (Good2Go, McGill library, McMaster…) and do not use non-validated information from internet.* | Yes | 3 | 2 | 3 |
| *69* | Shall we setup the application to work with AR (Augmented reality) such as with Samsung glasses? *If so, as the device is expensive, we may study its viability.* | No | 1 | 7 | 0 |
| *70* | Do we integrate the app with other applications already available? *If so, please list them at the comments on the last page. We may need to consider that they are in compliance and adequate for the audience.* | Yes | 6 | 1 | 1 |
|  | **General Questions - GAMING** |  |  |  |  |
| *71* | In your opinion, will the gaming help on engage the user? | Yes | 7 | 1 | 0 |
| *72* | If yes, shall we incorporate gaming to the application for this first version? | Yes | 4 | 1 | 2 |
| *73* | Do we reward the user upon the points gained versus other users? *e.g., Monthly competition.* | No | 2 | 5 | 1 |
| *74* | Do we reward the patient upon the points gained outside of the application? *e.g., Free tickets for a* Circle du Solei *exhibition.* | No | 1 | 3 | 4 |
|  | **Now consider different levels of gaming…** |  |  |  |  |
| *75* | OPTION 1: Application with the *appearance* of a game (Journey). Do we ONLY use the gaming as a “*fill conducteur*”? *Journey or Maze look and feel but the modules will not have games or activities but educational information (videos, PDFs, images and questions).* | Yes | 5 | 3 | 0 |
| *76* | OPTION 2: One game with different levels of complexity. Would it be engaging? *Imagine we have a UNIQUE game those stages (levels) get open as a reward every time the user completes a module or session. The more the user learns (completes modules) the more stages he can play.* | Yes | 5 | 3 | 0 |
| *77* | OPTION 3: Multiple activity games. Must each module have a specific game or activity? *Imagine we have SEVERAL games. It will certainly increase the scope of the HIT.* | No | 0 | 8 | 0 |
