# Supplementary material for: Development of MyREADY Transition BBD Mobile App, a Health Intervention Technology Platform, to Improve Care Transition for Youth With Brain-Based Disabilities: User-Centered Design Approach
Source: JMIR Pediatr Parent. 2024 Oct 1;7:e51606. doi: 10.2196/51606 (PMC11480690; doi:10.2196/51606)
Supplement: Multimedia Appendix 2 [file pediatrics_v7i1e51606_app2.pdf]

Supplement Table 1. Technology development and validation: user-centered agile design iteration flow.

Activity		Description
**Stakeholder knowledge exchange**		
	Methods used	• Facilitate discussions narrating patient needs, preferences, and desires.<br>• Identify common challenges in transition of care.<br>• Interactive stations to test and understand the utility of current HIT[a] tools.<br>• Stakeholder knowledge exchange question set provided on page 7-8.
	Selected results (key emerging themes)	• Integration of app into health care<br>• Transition is a holistic concept<br>• Keeping the technology simple<br>• Provision of education<br>• Empowerment<br>• Dynamic rather than static technology<br>• Strength-based approach
	Selected iterative app adjustments	• Feedback used to guide the early development of the eHealth tool.<br>• A key action identified was to develop a PFAC[b] to partner with us throughout development.
	Example of feedback not incorporated	• Feedback that was not aligned with the project objectives and scope (eg, transition in broader areas of life such as employment and school) were not incorporated.<br>• Some feedback was deferred for future versions of the app. For example, there was a suggestion from PFAC to add chat channels for the participants to be able to share experiences with one another. As this required the recruitment of a chat channel moderator and was beyond the study budget, it was deferred for future app development.

**Participatory design**		
	Methods used	Participatory design to define values, prioritize ideas, solutions, and content; contextualize solutions as part of daily life. 26 PFAC advisory meetings held throughout development. PFAC partners collaborated on key discussions and decision-making, for example:<br>• Sharing preferences for content, video, and design elements.<br>• Reviewing app content outline.<br>• Codeveloping focus group and interview questions.<br>• Field-testing app beta version.<br>• Generating content for activities in the app by providing examples from lived experience.<br>• Reflecting on the extent to which app storyboards and video scripts support learning goals in the app.
	Selected results	Example *results of PFAC review of app content outline.*<br>New content themes were suggested in the feedback:<br>• Anxiety and feeling anxious (normalizing, some will feel anxious, some will not, offering ways to practice, and help ease the anxiety or stress)<br>• Advocating "becoming an expert on yourself," the more youth understand about themselves, the better equipped they will be to advocate and get the right help for themselves. (communication skills, being prepared with questions, finding a good match with health care provider, and choosing someone to advocate on your behalf when necessary)<br>• Transitioning is a gradual process; there is value in seeing the "big picture" game plan and how it can be broken down into small workable goals (eg, weekly), doing individual goal setting. Establishing goals and routines is great, but also need to find ways to motivate them to stick to it.<br>• Troubleshooting the complexities of the adult medical system (eg, what to do when there is no one to go to?).

		• Balancing health with other aspects of life, reinforcing that the reason to be healthy is so you can fully participate (while this app is mostly about health care we do recognize how school/social/medical aspects are intertwined).<br>• Using language that is "less medical" and more strengths-based (eg, asking them to describe their strengths and weaknesses vs describing their condition).
	Selected iterative app adjustments	Example: early PFAC conversations provided advice on the design of the app:<br>• Many liked the proposed idea of a "city" layout. It was expressed that a city design could help to keep the modules neatly compartmentalized and keep the app from looking/feeling too juvenile and could support long-term sustainability of the app if it was to be broadened to include more than just health care transition (eg, social and educational transition).<br>• PFAC comment: "it's nice that it is sequential and parallel to the journey that is life."
	Example of feedback not incorporated	Incorporating content outside of the health care such as transition in education (eg, college or university), employment, or sexual education.
**Focus group**		
	Methods used	Focus group to gather opinions on main features and proposed content/educational modules. Focus group question set is provided on page 9-11.
	Selected results	Example results for question, *How do you feel about the ORDER OF THE MODULES?*<br>• When a user is first being introduced to the app, seeing the entire city and all the buildings might be overwhelming and look like a lot of work.

		• Focus group participants suggested starting with a few buildings and then “unlocking” new buildings as the user progresses through the modules/journey.<br>• Focus group participants wondered about the possibility of tailoring the order of buildings/content based on the user’s answers to questions at the beginning to zero in on what is most relevant to them at the time. They talked about the idea of the app “growing” with the user.
	Selected iterative app adjustments	Consolidating prior feedback with focus group results,<br>• the research team prepared on a beta version of the app<br>• in each chapter, the user visits a building with related content.
	Example of feedback not incorporated	The request for random selection of module-based content based on personal choice. This feedback was not incorporated because of the need for sequential content of the cognitive curriculum where each module builds from the previous one.
**Formative usability test**		
	Methods used	Qualitative interviews and observations of app beta version test to optimize the user interface and feature integration; assess prototype’s strengths and weaknesses; assess patients’ likes and dislikes.<br>• Beta version of the app was downloaded and installed onto a phone and/or tablet for the youths and their parents to try out.<br>• Participants were instructed to “think aloud” as they played with the app; the first 30 minutes of the interview involved observation by research staff, followed by interview questions.<br>• Formative usability test: qualitative interview question set is provided on page 12-13.
	Selected results	Example results for question 5: *What did you think about the activities in the App (e.g., videos, puzzle, etc.)? Did you find these helpful for understanding the content? Did working through the activities motivate you at all to learn more about transition?*

		• Older youth learn by first memorizing definitions and then by seeing what it means and how it applies in real world situations.<br>• Younger youth learn by seeing a visual first, we show them and then we explain it. The users of this app are in between those 2 age groups.<br>• Words need to be defined through experience for youth with BBD. Otherwise, it is like being at school and learning vocabulary.<br>• Suggest that videos/testimonials always appear before definitions/vocabulary. (eg, 1B Scene3 and Scene4 should come later or could be removed).
	Selected iterative app adjustments	Formative usability testing results informed next iterations to arrive at a full version of the app for summative usability testing:<br>• Generation of App content for psychology and informational content team<br>• Storyboard and video scripts review<br>• Video preferences survey<br>• Full review of app content
	Example of feedback not incorporated	Rating the videos individually and sharing such rating with other participants. Although the spirit of this idea was appreciated, at this stage, the app does not enable content sharing based on privacy consideration of the user community.
**Summative usability test**		
	Methods used	Quality assurance: effectiveness, efficiency, ease of use, user learning; measuring user experience; minimizing user errors<br>• Youth, parents, and health care providers download and install app; test for 10 days; complete worksheet and questionnaire; share experience in an interview.<br>• Summative usability test: interview question sets are provided on page 14-15.

	Selected results	Example results for *Overall how easy was it for you to move through from the beginning to the end of the app? Did you have any difficulties? Please describe:*<br>• Several users had problems with navigating section 1A of the app, specifically not knowing to click on the "Welcome to MyCity" sign and then being able to wander to different parts of the city, which left them feeling "lost" and not knowing what they should be doing next.<br>• Another navigation problem identified in section 1A was a glitch with buttons not appearing in the correct order, and users having to search for where to click "next."
	Selected iterative app adjustments	Critical fixes were prioritized to have a trial-ready version of the app.<br>• The navigation concern in section 1A was prioritized as a fix before starting the randomized controlled trial.<br>• The programmers were aware of the "where to click next" glitch and were already working on a "What's Next" button.<br>• The orientation session was improved for intervention participants: the RA[c] will assist with app download, guide through session 1A, and show the support website.
	Example of feedback not incorporated	The possibility of adding to the "What's Next" click with a "Go-to" option to improve automated navigation within the app. We declined the request because the programming burden was excessive at the point when the request was made.

[a]HIT: health information technology.

[b]PFAC: patient and family advisory council.

[c]RA: research assistant.

STAKEHOLDER KNOWLEDGE EXCHANGE QUESTIONS

Stakeholders involved

19 stakeholder attendees: 1 Cardiologist, 1 Research Consultant with expertise in Health Information Technology, 2 Developmental Pediatricians, 2 Occupational Therapists, 3 Youth (2 with a disability: chronic health condition; hemiplegia), 2 Trainees, 2 Nurse Practitioners, 2 Child Life Specialists, 4 Research Staff.

Interactive Station Questions

Attendees rotated between the three different stations, spending approximately 10-15 minutes at each station, and answering the questions in the table below:

1. Have you ever used online programs or tools/apps like this on your phone or tablet? PROBE: If yes, what was your experience? If no, why?
2. What is your overall opinion about this tool? PROBE: What do you like the most about it? What do you like the least about it?
3. In general, do you think its functions are useful? PROBE: Do you think anything should be added? Do you think anything should be taken out?
4. In general, do you think that you will use this tool? PROBE: How easy or difficult do you think it would be to use it?
5. Do you have any suggestions for how we can change or improve this tool?

Large Group Brainstorm Questions

**Questions about the proposed online tools/programs (20 minutes):**
1. In general, what is your overall opinion of these online tools or programs?<br>PROBES:<br>• How can they successfully help users in their transition (if at all)?<br>• Which tools and features you think would be useful for you? Why?<br>• What technology are you currently using most & how does that impact which tools you would prefer to use? (e.g., phones, tablets, computers, apple, android)<br>• What topics do you think are the most important to cover in the tools?

2. Do you have any suggestions for how we can change or improve these tools?<br>PROBES:<br>• What would you think about the different tools you saw today being available on one single platform?
3. Based on your experience, how easy or hard it would be for you to use your phone/tablet/computer to operate these tools?
4. Based on your experience, how involved would you like your healthcare provider (e.g., your doctor) to be in your transition management program?<br>PROBES:<br>• Would you like to communicate with your healthcare provider via these online tools/programs?<br>• Would you prefer to contact your healthcare provider directly if you need additional assistance or have questions?<br>• Would you like your healthcare provider to receive information from these tools?
**Additional General Questions (30 min)**
5. In your own opinion, what makes a transition management program successful?
6. What do you think patients and families want in an intervention?
7. What are the priorities of patients/families?
8. What are the gaps you think can be addressed in a stand-alone IT intervention? (e.g., vs. changing the health care system and its challenges)
9. How would you recognize that transition planning is happening or progressing (i.e., early, middle, and late stages)?
10. What would transition success look like from a patient/family perspective?
11. What words do you use around the topic of transition? (is it different to our jargon?)
12. Is there anything else that you'd like to add or tell us related to anything that we talked about today?

FOCUS GROUP QUESTIONS

Stakeholders involved

6 research participants: 4 youth/young adults: 1 female, 3 male, aged 18-23 years. 2 parents: 1 attended the focus group. 1 interviewed at a later time. Collective lived experience of acquired brain injury and cerebral palsy.

I. GENERAL QUESTIONS (5-10 min)

The first set of questions will ask about your experiences with anything you have tried for taking charge of your own health to improve your health care or transition from youth to adult care.

1. What types of things have you (or your child/patient) used to try improving your (their) transition from youth to adult health care?
    a. PROBE: If it was helpful – why do you think it was?
    b. PROBE: If it was not helpful – why do you think it was not?
    c. PROBE: How have you put into practice any of the ideas from _____?
2. Have you (or your child/patient) ever used online programs or tools, such as websites or apps on your phone, tablet or computer **related to your health condition**? Let's talk about your experiences.
    a. What kinds of things make a good health care website or app?
3. Now let's talk about whether you have ever looked online for apps or information about the **transition from youth to adult care**?

II. ONLINE PROGRAM QUESTIONS (10 min presentation plus 30 minute discussion)

Our goal is to provide an app that can help youth between 15 and 17 years old with brain-based disabilities to become more in charge of their health when transitioning from pediatric to adult health care systems – for example, doing things like booking their own appointments and answering a doctor's or nurse's questions. We have developed something that we are currently calling Teen Town, in which users will be able to move between buildings during their health care transition journey, and along with their Coach who is the main character of the game. There is a "home base" which is meant to be the entry point for the app – where you'll start from each time you open it up to use it again. Other buildings include a house, hospitals and a school.

I will start by sharing some visuals to give you an idea of the main features of the app and asking you what you think.

1. What is your overall opinion of the Teen Town concept?
    a. What do you like the most about it?
    b. What do you like the least about it?
    c. How do you feel about the name Teen Town? Is there another name you can think of that might appeal to youth? (PROBE: MyCity?) How do you feel about users getting to name the city themselves?

2. In this app users journey with their “Coach” through Teen Town and carry out activities in different settings like rooms and buildings. Based on what you’ve seen so far:
    a. Are there any particular settings you would change, or ones we’ve forgotten to include?
    b. How do you like the term “Coach” to describe the person who helps the user along the journey? In what ways would you like to customize the Coach? (PROBE: clothing, accessories, hair, etc.)
    c. What do you think would be a good name for the user’s “home-base”? (PROBE: Clubhouse, Transition Centre/Club, Recreation Room?)
    d. Is there anything else you’d like to tell me about Teen Town or its design?

III. EDUCATIONAL MODULES (10 min presentation plus 30 minute discussion)

Now we will share with you about proposed educational topics to be addressed in the app. All of this content is aimed to improve youth’s knowledge and self-management skills. The idea is that you learn about taking charge of your own health by doing things like watching videos, doing challenges or quiz games, and learning from a “coach” who is your companion throughout Teen Town.

I will start by sharing some visuals to give you an idea of the educational modules and asking you what you think.

Module Questions

1. What is your overall opinion of this content?
    1. Are there parts (e.g., content, tasks/challenges) of the modules that stand out to you as being more useful in getting ready for transition?
    2. Are there any parts that seem less useful?
    3. How do you feel about the order of the modules?

2. A major part of the app will include watching short video clips in which users can learn about a transition topic. What style of video do you think appeals most to youth? (e.g., selfie-style, animation,YouTube, Actors)
    i. PROBES: How long should the videos be?; Who do you think should be the actors/characters in the video (e.g., youth, youth with disabilities, health care providers)?; Should they be the same or different actors throughout?; Would you prefer the same style of video throughout, or a mix of different styles (e.g., actors and animated videos)?

3. We want to create an app that is useful and that will be interesting enough for people to use it.<br>1. How do you feel about earning points during learning activities to use for either playing games or further customizing your Coach?<br>2. How do you feel about the different game examples shown here?<br>4. Do you have other ideas for what might motiviate people to use an app like this?
5. One of the activities we plan to include in the app involves helping youth better understand the differences and similarities between pediatric and adult hospitals. Based on your experiences, what would you describe to be the main similarities and differences?<br>i. PROBES: if needed, provide an example
**OTHER (If time permits)**
6. Is there anything else you would like to add that might be important for us to know?

FORMATIVE USABILITY TEST – QUALITATIVE INTERVIEW QUESTIONS

Stakeholders involved

6 research participants: 4 youth/young adults: 2 female, 2 male, aged 15-23 years. 2 parents (mothers). Collective lived experience of autism spectrum disorder, cerebral palsy, and spina bifida.

Observation (30 MIN)

The first thing I'd like to ask you both to do today is to play around with our early version of the application on both an iPhone and an iPad. You'll have 10-15 minutes to use one version and then we'll switch and try the other. As you use the application, feel free to think aloud and share your experiences with us and with each other – for example, things you like about the app, things you're struggling with and need help, etc. – this is all very important for our team to know. When you're finished I have some questions that I'll ask you about what it was like to use the application. Does anyone have any questions before we get started?

**I. USER FEEDBACK (35 MIN)**<br>Now that you've had a chance to try out the application, I'd like to ask you a few questions about your user experience.
1. In general, how easy or difficult was it to use the application? [probe: on iPhone? on iPad?; any differences between the two?]
2. If you were going to use this application, would you imagine yourself using it on a phone, a tablet or a desktop computer? Why?
3. How easy was it to navigate yourself throughout the application? Did you encounter any challenges? Please describe.
4. How easy was it to understand the information being presented to you in the app?
5. What did you think about the activities in the app (e.g., videos, puzzle, etc.)? Did you find these helpful for understanding the content? Did working through the activities motive you at all to learn more about transition?
6. Now that you've had a chance to play with the app, how do you feel about whether or not it was a good use of your time? [probe: What can someone gain from using the app? Would be the app be helpful during transition? Is the app something you think you would use?]
7. What other recommendations or comments might you have for us? [probe: if you could improve anything about the app, what would it be? E.g. design, content, technical features]

**II. CONTENT GENERATION FOR PSYCH TEAM (25 MIN)**<br>The final set of questions that I have for you today come from the team who is building the content for the application. They're looking for help from patients and families to generate ideas for the application – related to some of the activities, challenges and information presented throughout.
1. In one activity, the user is presented with some of the general differences and similarities between pediatric and adult hospitals. What can you come up with that would be good examples of how these hospitals differ? Are the same?
2. In another activity, the user is provided with reasons why a young person might be either excited or scared to enter into adult healthcare. If we wanted to make this meaningful to someone like you, what is/was your experience? What makes/made you excited? What makes/made you feel afraid?
3. We also want to create a list of things that young people might want to know when making decisions about their health (e.g., questions about medication, exercise). What suggestions do you have that we could add to this list?<br>What about the types of questions that a young person might have during their last visit with a pediatric health care doctor – so before transitioning to adult care?
4. Within the app, there will also be an activity about things young people need to do every day to take care of themselves. If you were asked to make a list of the every-day things you do to take care of yourself, what would you include?
5. Finally, the app includes information on developing independence from parents. What ideas do you have for ways that youth can start doing things on their own without their parent(s)?

SUMMATIVE USABILITY TEST – INTERVIEW QUESTIONS

Stakeholders involved

12 research participants: 8 youth/young adults: 2 female, 2 male, aged 15-21 years. 2 parents (1 mother, 1 father). 2 health care providers (occupational therapist, nurse practitioner). Collective lived experience of acquired brain injury, autism spectrum disorder, cerebral palsy, epilepsy, fetal alcohol spectrum disorder, and spina bifida.

For Youth/Young Adults:

**GENERAL QUESTIONS**<br>1. What device did you use the app on? (desktop or laptop computer, tablet, phone)? Did you use more than one device? Why?<br>2. What device did you prefer to use? [probe: what made the experience different?; did you have any concerns about using it on a particular device?]
**REVIEW OF WORKSHEET –** briefly review participants' written feedback
**A. DESIGN**<br>1. **Layout**: Did you find that the City design was helpful for organizing the information you learned about in the app?<br>2. **Navigation:** Overall how easy was it for you to move through from the beginning to the end of the app? Did you have any difficulties? Please describe.<br>3. **Visual Appeal**: How did you feel about the look of the app? [probe: illustrations, graphics, animations, icons, size of screen, font]<br>4. Do you have any other suggestions or comments for us about the design of the app?
**B. CONTENT**<br>1. **Completeness**: Did the app contain everything you would expect it to, to help prepare someone for transition?<br>2. **Understandability**: How easy was it to understand the information in the app? [probe: were things worded in a way that you could understand?]<br>3. **Relevance** Did you feel that the information was okay for youth with different abilities?<br>4. Do you have any other suggestions or comments for us about the information included in the app?
**C. FUNCTIONALITY AND FEATURES**<br><br>1. **Mentor:** What did you think about the Mentor? Did you find the mentor useful while going through the app?<br>2. **Activities:** What did you think about the activities in the app (e.g., games/challenges, puzzles, pop-up questions, sorting/matching activities, drag & drop etc.)? Did you find

these helpful for understanding the information? Did you find these were too long/too short? Did you have difficulties with any of these activities?

3. **Bulletin board:** How helpful was the bulletin board? Did you return to it at any time when you were using the app?
4. Do you have any other suggestions or comments for us about the mentor? the activities? the bulletin board?

D. VIDEOS

1. What did you think about the videos in the app? Did you find these helpful for understanding the information? Did you feel these were too long/short?
2. Do you have any other suggestions or comments for us about the videos?

E. REWARDS

1. What did you think about the rewards in the app? (e.g., coins, plaques, trophies). Did you use the coins in the arcade? Did the rewards make you want to keep going?
2. Do you have any other suggestions or comments for us about the rewards?

F. DESIRE TO USE PROGRAM IN THE FUTURE

1. Now that you've had a chance to use the app, how do you feel about whether or not it was a good use of your time? [probe: What can someone gain from using the app? Would the app be helpful for someone getting ready for transition? Is the app something you think you would use? Would you recommend it to others? Who?]
2. In general, what other suggestions or comments might you have for us? [probe: if you could improve anything about the app, what would it be?

For Parents, Healthcare Professionals:

REVIEW OF WORKSHEET – briefly review participants' written feedback

Questions:

1. Now that you've had a chance to use the app, do you think it would be helpful for someone getting ready for transition?
2. Did the app contain everything you would expect it to, to help prepare someone for transition?
3. Did you feel that the information in the app was okay for youth with different abilities?
4. Is the app something you would (or would have) recommend(ed) to your child/your clients?
5. Do you have any other suggestions or comments for us about the app [probe: design, videos, information/content, rewards]?
