# Supplementary material for: Development of MyREADY Transition BBD Mobile App, a Health Intervention Technology Platform, to Improve Care Transition for Youth With Brain-Based Disabilities: User-Centered Design Approach
Source: JMIR Pediatr Parent. 2024 Oct 1;7:e51606. doi: 10.2196/51606 (PMC11480690; doi:10.2196/51606)
Supplement: Multimedia Appendix 3 [file pediatrics_v7i1e51606_app3.docx]

**Supplemental Table 2.** Description of MyREADY Transition BBD app’s key customization and personalization features.

| Feature | Description | Customization | Personalization |
| --- | --- | --- | --- |
| Mentor appearance and accessories | The main character is a mentor or companion that guides the user in the adventure.  *(eg, an Indian male with crutches in a blue T-shirt, wearing a red hat and sunglasses)* | - 12 choices of mentor character with different options for ethnicity and gender/nongender defined. - 19 choices of accessories including crutches, clothing, sunglasses, and other gender-neutral objects. | - The user can change the mentor at the beginning of each section. - After completing a session, the user unlocks new accessories to add to the wardrobe. |
| Text-to-speech, sounds, and volume control | The mentor has a voice that can be customized to match other features such as language or gender.  *(eg, Text-to-speech option delivered by the mentor who reads the screen messages)* | - Text-to-speech option for mentor text added as an accessibility feature. - Mentor voice options carefully selected to be clear and realistic. - Extra pauses coded to enhance the text-to-speech feature, delivering a more natural versus robotic voice. - Other in-app sound effects and music added to enhance user experience. | - The text-to-speech and in-app sounds can be customized according to the user’s preference (eg, choosing mentor voice and language, changing or muting the volume of sounds). |
| Short videos with subtitles | The app contains up to 135 videos (47 conceptual and 88 testimonial). *(eg, At the beginning of each session, the key points of the previous session are summarized in a conceptual video)* | - All testimonial videos contain captions in English or French as an accessibility feature. - Conceptual videos use visuals and written text to reinforce the messaging. - Average video length is 30-90 seconds as a strategy to retain user attention. | - The user can choose their preferred language for subtitles. |
| Progress indicators | The user can see a visual representation of how much they have completed already relative to the total content. *(eg, At the top of the screen, the user can see the progress bar for the current session filling up as they play)* | - A progress bar shows the user their progress throughout each session and video. - At the end of each session, the user earns a plaque, and at the end of each chapter, a trophy for their trophy case. The rewards in the trophy case also serve as a reminder of overall journey progress in the app. | —^a^ |
| User navigation features | The “What’s next” button and blinking items lead the user to the location of the next activity and remind them of what they are expected to do next. *(eg, When the “The Health Clinic” is next in the journey, the “What’s next” message will serve as a reminder and the building will be blinking in the City)* | - As an accessibility feature, a button to cue the user and help them find the next action and location was added. Blinking objects also visually highlight the area where the user should click next. - Thumbnails (shortcuts) to MyCity buildings across the bottom of the screen add to ease of navigation. | - The “What’s next” button is an optional feature that can be accessed on an as-needed basis. - The user can choose to disable the thumbnails (shortcuts) to the MyCity buildings. |
| Color contrast and animation | In keeping with gaming design, the app uses color contrast with no shadows and animated elements. *(eg, The city has 2 main streets with colored buildings that are well organized and labeled. Animated pedestrians, bus and cyclists create a lively city scene)* | - Bright colors create an engaging and high-contrast learning environment. More subdued colors were used in the menus and tabs (eg, About the App) to create visual distinction. - Some elements in the app are animated using basic movements to make the learning environment more dynamic. - The app itself is based on the concept of “moving forward in the Journey.” | — |
| Secure server | To protect user confidentiality, all user data are anonymized and stored on a secure server. | - For users enrolled in the READYorNot BBD^b^ trial, the secure servers are based in Canada following the PIPEDA^c^ and provincial standards. | - Data stored locally on the user’s device and transferred to the secure servers at the end of each session. |
| Detailed technical support resources | FAQ^d^ section in the app, support website (with download links), and technical support team provided to users enrolled in the READYorNot BBD trial. | - All the resources available in English and French. - Ticketing system used by research staff to track and follow up on user support issues identified through the support website. | - Users can use FAQ and tutorial videos to troubleshoot on their own and/or can request technical support through the website. |
| Content available in plain language English and French | The app’s user-facing content is available in plain language English and French to be consistent with both official languages in Canada. | - The app is provided in both official languages of Canada to be inclusive. - Readability of text is tailored for accessibility (eg, using plain language and simple sentence structure). | - Users can choose their preferred language either at initial app setup or between sessions. |
| Offline mode | Some app content uploaded during the app installation is available when the app is offline. | Some offline capability was included to provide limited app functionality at times when the user does not have internet access. | When the app is used offline, videos and text-to-speech features cannot be accessed, and the app is not synchronized with the content management system. |

^a^Not applicable.

^b^READYorNot BBD trial: Readiness in Youth for Transition Out of Pediatric Care brain-based disability trial.

^c^PIPEDA: Personal Information Protection and Electronic Documents Act.

^d^FAQ: frequently asked question.
